# Zoonotic Risk: One More Good Reason Why Cats Should Be Kept Away from Bats

**DOI:** 10.3390/pathogens10030304

**Published:** 2021-03-05

**Authors:** Valeria B. Salinas-Ramos, Emiliano Mori, Luciano Bosso, Leonardo Ancillotto, Danilo Russo

**Affiliations:** 1 Wildlife Research Unit, Dipartimento di Agraria, Università degli Studi di Napoli Federico II, Via Università 100, 80055 Portici, Italy; valeria.salinasramos@unina.it (V.B.S.-R.); leonardo.ancillotto@unina.it (L.A.); 2 Consiglio Nazionale delle Ricerche, Istituto di Ricerca sugli Ecosistemi Terrestri, Via Madonna del Piano 10, 50019 Sesto Fiorentino, Italy; emiliano.mori@cnr.it

**Keywords:** bat, cat, COVID-19, SARS-CoV-2, spillback, spillover, zoonotic risk

## Abstract

Bats are often unfairly depicted as the direct culprit in the current COVID-19 pandemic, yet the real causes of this and other zoonotic spillover events should be sought in the human impact on the environment, including the spread of domestic animals. Here, we discuss bat predation by cats as a phenomenon bringing about zoonotic risks and illustrate cases of observed, suspected or hypothesized pathogen transmission from bats to cats, certainly or likely following predation episodes. In addition to well-known cases of bat rabies, we review other diseases that affect humans and might eventually reach them through cats that prey on bats. We also examine the potential transmission of SARS-CoV-2, the causal agent of COVID-19, from domestic cats to bats, which, although unlikely, might generate a novel wildlife reservoir in these mammals, and identify research and management directions to achieve more effective risk assessment, mitigation or prevention. Overall, not only does bat killing by cats represent a potentially serious threat to biodiversity conservation, but it also bears zoonotic implications that can no longer be neglected.

## 1. Introduction

The ongoing COVID-19 (COronaVIrus Disease 2019) pandemic has highlighted the primary role of wildlife in zoonotic events (e.g., [1]) that due to the high density and mobility of the human population can spread rapidly over large regions of the globe.

While much of the public attention has focused on the epidemiology of SARS-CoV-2 as a human pathogen, there is little doubt that the causal factors that originated the spillover lie directly or indirectly in the consumption of bats by humans, frequent in Asia [2]. More generally, there is consensus on the fact that zoonotic diseases are strongly favored by the ever-growing deforestation and expansion of farmland and urban areas at the expense of natural habitats, increasing the opportunities for wildlife–human interactions, as well as for wildlife traffic and consumption [3]. It is therefore clear that a holistic approach to the prevention of zoonotic diseases taking into account humans, wildlife and environmental socio-ecological dynamics is necessary to prevent future spillover processes and pandemic events.

The zoonotic risk associated with wildlife is often multifaceted and may involve several actors. Therefore, only accurate surveillance of all the species that take part in the human–wildlife interaction network may indeed lead to effective prevention and mitigation. Domestic animals may play a crucial zoonotic role in bridging wildlife and humans in situations where direct human–wild animal contact would otherwise be rare, amplifying the pathogen or acting as a vessel for genetic variation [4]. To mention one example, in the case of the Nipah virus in Malaysia, domestic pigs acted as amplifiers of the virus carried by fruit bats and passed it on to humans [5].

Bats are often mentioned as species characterized by a high zoonotic potential ([6]; but see [7]) and natural reservoirs of pathogens (especially viruses) that may affect humans directly or after a more complex pathway involving other animal species. While the risk posed by bats is overwhelmed by the vital ecosystem services these mammals provide, and misrepresentation of such zoonotic risks may harm bat conservation [8], an often-overlooked argument is that direct contacts between bats and people are relatively unlikely if bushmeat or human encroachment on bat habitat does not occur. Bats being nocturnal, elusive mammals that typically avoid contact with humans and are highly sensitive to anthropogenic disturbance, such contacts are in fact very rare. Nonetheless, predation on bats by domestic animals which have frequent contact with humans might represent an important epidemiological link so far only partly explored.

**Figure 1:**
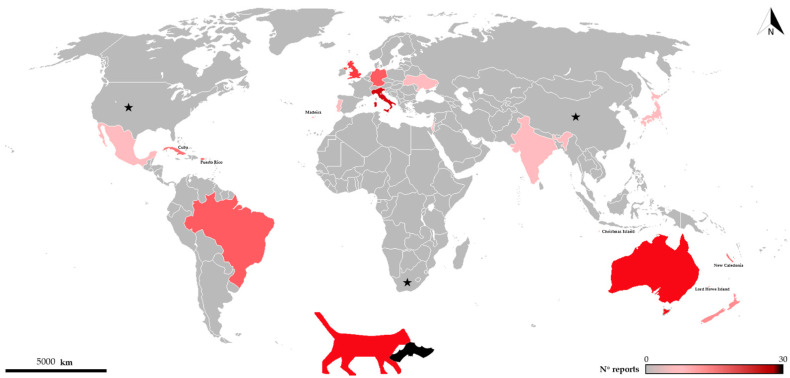
Distribution of records of bats killed by cats throughout the world. Color intensity increases with number of records. Black stars show countries where predation on bats by domestic cats is reported but number of records/species is not available [11,12].

As documented worldwide, domestic cats are well-known predators of many wildlife species [9], sometimes in ecologically vulnerable systems such as islands [10] where their impact may be especially pronounced. As for bats, cases of predation by domestic cats cover 48 bat species and ca. 20 countries (see Figure 1 and Table 1), and due to the difficulties in recording such events, there is no doubt that this phenomenon is much more widespread than previously imagined.

For instance, cat predation was found to be the first cause of admittance to Italian wildlife rescue centers for adult bats [13].

While the actual population effects on wildlife of cat predation are unknown, yet suspected to be important, there is overall little doubt that where bats and domestic cats co-occur, this typically results in predation episodes (see Table 1 and references therein). The topic is often discussed in terms of conservation consequences since many bat species are at risk, and cat predation may at least act synergically with other threats contributing to jeopardize bat conservation status, especially when already dwindling small bat populations are involved.

In general, obligate zoonotic pathogens are usually transmitted from an animal to people (in which case, the latter normally receive the pathogen from animals). Human pathogens, on the other hand, are typically passed on from human to human, but when they have an animal reservoir, they may also be transmitted from an animal to a person. Finally, an animal pathogen may evolve and adapt to our species, becoming a new human pathogen which is then passed on predominantly from human to human. In the last case, the novel human pathogen might still be transferred from people to an animal reservoir, which represents a further way of transmission to humans when it comes into contact with the latter.

**Figure 2:**
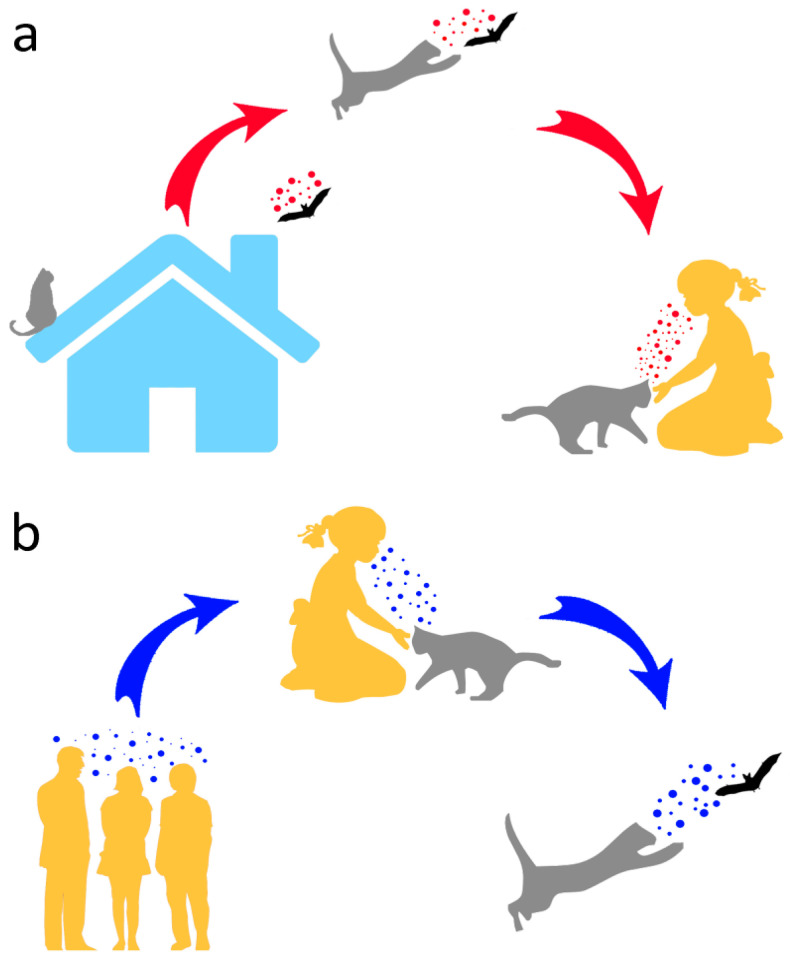
Potential human–cat–bat interactions and associated pathogen transmission. In (**a**), pathogens are transmitted by a bat caught by a cat, and eventually to humans from the latter. In (**b**), a reverse pathogen transmission from humans to bats via cat predation is shown.

Therefore, in addition to posing conservation problems, as opportunistic predators of wild animals, domestic cats may help to open the Pandora’s box of wildlife (including bats) pathogens by (a) contracting such pathogens from their wild prey and transmitting them to humans and (b) providing a further evolutionary environment that might ultimately lead to diseases affecting humans or other animals (Figure 2). Moreover, due to their frequent interactions with people, typically involving physical contact, cats might host human pathogens and pass these on to bats (Figure 2).

Because of their peculiar physiology and natural history, bats are highly resistant to viruses [46], so in the worst-case scenarios, the above-described interactions might make bats viral reservoirs of pathogens that in the absence of predation-mediated transmission would never reach them. In addition to being species of conservation concern strictly protected in most countries (in the EU, all bat species and their habitat are protected under the 92/43 Habitats Directive), bats provide vital ecosystem services such as insectivory, pollination and seed dispersal [47,48], and insect suppression by bats is in fact an important natural defense from arthropod-borne diseases. Therefore, should bats become a novel pathogen reservoir, culling bats even under hypothetical emergency scenarios must be firmly ruled out as a solution as it would surely have detrimental consequences for ecosystem functioning, human economy and health.

## 2. State of Art of Bat–Cat Exchange of Pathogens

### 2.1. Rabies

The best-known disease that may pass on from bats to cats and eventually humans is rabies, a zoonosis that causes 590,000 human deaths per year and affects >100 countries worldwide [49]. Human contagion is caused by contact with saliva of an infected animal. Cases of Rabies Lyssavirus (RABV) in the Americas are often associated with bats [50,51], and the virus was also recurrently isolated from domestic cats. For example, in 2007 alone, 3.8% of RABV cases reported for the US and Puerto Rico were cats (*n* = 274; [52]). In the same geographic area, in 2018, cats were 4.9% of 4951 cases of animal rabies [53]. Lyssaviruses other than RABV such as European Bat Lyssaviruses (EBLV) types 2 and 3, also potentially fatal to people yet with extremely low human incidence across Europe, have bats as the main host and have been also rarely isolated from domestic cats in the European territory [54]. Contacts between domestic cats and rabid *Eptesicus serotinus* bats were reported for the Netherlands [55]. Most recently, a cat in Central Italy died after biting humans and tested positive for another lyssavirus, the West Caucasian bat lyssavirus [49], previously isolated in the *Miniopterus schreibersii* from South-Eastern Europe [56]—a partly migratory species also occurring in Italy [57]. Isolates of the African Mokola virus (genus *Lyssavirus*) are also retrieved in domestic cats and might result from predation over a bat or rodent natural reservoir [58].

Although in many cases, cats are likely to be infected from other wildlife such as carnivores, this picture still points at a non-negligible risk of spillover events from bats to domestic cats [59], which has in fact been observed [60]. Although cats seem to be resistant to acquiring rabies through the ingestion of contaminated tissue [61], they may still be exposed to (and infected through) bat bites when capturing bats on dusk emergence from the roost. Due to its altered movement and reduced reactivity, a rabid bat fallen to the ground in the surroundings of its roost [59] may be especially attractive to the predator [62,63]. While the risk run by humans of being bitten by a rabid wildlife species is small [64], this is intuitively substantially higher when it comes to domestic animals such as cats. Through this way, even historically rabies-free areas—particularly when involving migratory bat species [65]—and urban or rural environments [66,67,68] may be affected. For instance, in Brazil, in 2008–2016, only 1.4% cases of people bitten by animals concerned wildlife vs. 94% of bites by dogs and cats [64]. In the same country, variants of rabies viruses circulating in populations of vampire bats *Desmodus rotundus* were also isolated in domestic cats and transmitted to humans from both animal species [69]. Moreover, rabies may potentially be transmitted from cats to humans through scratches, which are common injuries associated with people–cat interactions [70]. Domestic cats may therefore represent a new spillover-spreading route [71] through which an otherwise negligible incidence of bat rabies transmission to humans is magnified.

### 2.2. Other Diseases

In addition to rabies, a range of other diseases may directly or indirectly involve cats, bats and humans. For instance, domestic cats have been found to be susceptible to henipaviruses and filoviruses isolated from bats and might therefore act as intermediate hosts in outbreaks of these pathogens, but important knowledge gaps exist on this issue ([72], for a review). Other viruses such as the *EfHV* (*Eptesicus fuscus* Herpesvirus, a Gammaherpesuvirus) may replicate in bat, cat and human cells [73]. Further, interspecies transmission involving bats, cats and humans may foster viral genetic reassortments, which may also take place in viruses that are currently pathogenic to humans [74].

Gram-negative bacteria from the genus *Pasteurella* are commensal of dogs’ and cats’ oral cavities and may occasionally infect humans through bites or scratches [75]. Among these, *P. multocida* was reported to infect and kill free-ranging vespertilionid bats, which typically die by septicemia following pasteurellosis [33]. There is substantial evidence that *P. multocida* strains of cat origin were transmitted to bats following cat predation attempts [33], so even bats that survive the attack may develop wound infections and die afterwards. Other bacteria transmitted to bats in the same way that may also harm these mammals are those in the family Enterobacteriaceae [33]. Among protozoans, *Toxoplasma gondii*, a feline–zoonotic pathogen causing toxoplasmosis in humans to whom it is often transmitted by domestic cats, was also (albeit rarely) observed in bats, comprising genotypes previously isolated from cats [76,77]. Other protozoans harmful to humans such as *Trypanosoma cruzi* have a range of wild and domestic reservoirs, including bats and cats [78], yet no direct transmission between such species is documented. Yeasts that affect animals and humans such as the *Malassezia* spp., some of which cause skin disorders in people, are frequent on the skin and in the auricular canal of domestic cats and dogs and have also been detected in bats, suggestive of the hypothetic emerging zoonotic pathways involving, this time, fungi [79].

Arthropod ectoparasites are well-known vectors that transmit diseases to their hosts and can be directly involved in spillover events [80]. Although such parasites are often host-specific, some are opportunistic, such as the badger flea *Paraceras melis*, observed in several wild mammals, but also in dogs, cats, bats (*Rhinolophus hipposideros*) and humans [81]. The flea in question is the vector of *Trypanosoma pestanai*, affecting European badgers [82]. Likewise, mites such as *Demodex canis* may parasitize cats and dogs as well as bats [83] and rarely cause demodicosis in humans [84]. In such cases, arthropod ectoparasites might provide an epidemiological connection among different hosts and link humans to bats via cats, but this potential route is unstudied and warrants investigation.

## 3. SARS-CoV-2 and the Risk of Human-to-Bat Transmission. A Role for Cats?

Over the last few months, in the course of the (at the time of writing) still ongoing tragic pandemic, the media have too often presented bats as the epidemiological source of SARS-CoV-2, the virus causing COVID-19, framing these mammals in a negative light and exposing them to potentially adverse conservation consequences such as deliberate killing and roost disturbance [85]. In fact, rhinolophid bats from Southern China were found to host other coronaviruses (Bat CoV RaTG13 and RmYN02) that are phylogenetically strictly related to SARS-CoV-2, pointing at a common origin of this virus [86]. Although bats are therefore unfairly indicated as the culprit of the pandemic, the real causes are to be found in bat hunting and trading for human consumption [87]. Whether the SARS-CoV-2 evolution may have involved other hosts such as pangolins [88] is unclear and was recently questioned [89], but there is no doubt that live animal markets where bats, pangolins and other wildlife are stored together and in strict contact with people, slaughtered and sold in situ provide ideal conditions for the emergence of novel viral diseases [87]. Although COVID-19 is a human disease that had a zoonotic origin, research showed that SARS-CoV-2 spike proteins may bind to the ACE2 proteins found on the cell surface of several other mammals in addition to humans [90]. There have been, in fact, cases of non-human mammals infected with SARS-CoV-2 comprising captive tigers and lions, American minks in breeding facilities as well as in the wild, and pets (see [91] for a review). As for bats, the picture is unclear and far from being comprehensively explored. *Rousettus aegyptiacus* may be experimentally infected but show no symptoms [92], whereas other species, including *Tadarida brasiliensis* and rhinolophids, are deemed possibly susceptible to human-to-bat transmission [91]. On the other hand, big brown bats *Eptesicus fuscus* experimentally inoculated with SARS-CoV-2 proved resistant to infection [93]. Overall, reactions to the virus can be highly species-specific, which is of little relief considering the high global species richness of bats (>1400 species) and the widespread occurrence of SARS-CoV-2 among humans.

Based on the high diversity of coronaviruses found in bats and the tendency shown by such pathogens to change hosts [94], conservationists’ main concern is that transmission of SARS-CoV-2 from humans to bats (or other wildlife) would establish a novel reservoir in these mammals from which further waves of infection might be sparked (Figure 2). Should this happen, it would clearly constitute a very challenging managing issue with adverse consequences for bat conservation, especially in urban areas [91]. Moreover, by spreading in wildlife, including bats, SARS-CoV-2 might have the chance to evolve and adapt to a range of new hosts. On such a basis, in 2020, the EUROBATS Advisory Committee promptly published recommendations on potential risks of SARS-CoV-2 transmission from humans to bats (EUROBATS is the UNEP Agreement on the Conservation of Populations of European Bats: www.eurobats.org, accessed on 25 January 2021). In the same year, to prevent such risks, the International Union for Conservation of Nature Bat Specialist Group issued guidelines aimed at a diverse range of stakeholders including bat researchers, rehabilitators, cavers and cave visitors and guano collectors (https://www.iucnbsg.org/bsg-publications.html, accessed on 25 January 2021).

The presence of house-dwelling bats in their roosts is unlikely to pose a realistic risk of direct human-to-bat transmission of SARS-CoV-2; however, especially in rural or urban contexts, domestic cats may hypothetically bridge the physical gap separating people from bats and contribute to spread the virus to the latter through predation. People may in fact transmit SARS-CoV-2 to cats, which are susceptible to the virus and are subject to efficient (also airborne) transmission of the virus, as known from both recorded cases and experimental work [95,96,97,98,99,100]. While it is difficult to predict the likelihood of cat-to-bat transmission of SARS-CoV-2, which based on the scarce available knowledge seems negligible, there is no doubt that the issue merits full attention due to its considerable implications in terms of zoonotic risk and conservation consequences.

## 4. Preventing Zoonotic Risks Associated with Bat Predation by Cats: Research and Management Directions

From what has been illustrated so far, it should be clear that not only do domestic cats left outdoor pose threats to bat conservation, they also represent an epidemiologic link between humans and bats across which pathogens may spread in either direction. On such a basis, we urge that appropriate measures be taken to prevent the zoonotic risk associated with cats, especially in the proximity of known bat roosts, as follows:

1. Domestic cat populations should be part of active surveillance programs aiming at detecting the possible presence of pathogens that may be involved in the bat–cat–human transmission chain. The program should target cats left outdoor, which not only includes stray cats but also free-roaming owned cats;
2. In countries such as Italy, urban free-roaming cats living in colonies are protected by the law; cats are recognized the right to live free, neither killed nor moved, are neutered by the veterinary service of the local health authorities and are looked after by institutionalized cat carers [101]. While this degree of protection has certainly improved conditions of stray cats and pursued important animal welfare objectives, its consequences in terms of the impact on urban animal biodiversity are unknown and neglected. We propose these colonies be systematically monitored for their effects on wildlife, and more specifically bats, and the possible zoonotic consequences assessed to develop appropriate management strategies. Special measures should also be taken for cat colonies that occur in sites where a bat roost is known to be present;
3. Outdoor cats should be included as a threat factor in all conservation plans aiming at the management of known bat colonies, and action should be taken, including legal measures, to prevent contacts between cats and bats;
4. Where applicable, vaccination campaigns of cats living outdoor should be promoted to reduce major zoonotic risks such as that associated with rabies, and more investigation is warranted to see whether commercially available rabies–inactivated veterinary vaccines, prepared from RABV strains, can cross-protect cats against the different lyssaviruses circulating in Europe [102]. The risk of stopping vaccination campaigns in areas that have been recently declared rabies-free should also be evaluated [103];
5. Cat predation on wildlife is a complex process that is deeply rooted in cats’ evolutionary history but also depends on a range of variables including dietary requirements and physiological traits, early life history, individual personality and environmental factors [104]. A better understanding of the relative weights of such variables is key to develop appropriate strategies to prevent cat predation on wildlife and bats more specifically, and ad hoc work should be done on bat–cat interactions, including those involving sick bats. For instance, in a study on spatiotemporal trends in bat rabies in Washington State which assessed the associated zoonotic risk, cats caught almost 90% of bats captured by pets, but dogs tended to catch rabid bats more often [105]. Patterns such as this are clearly linked with the behavioral characteristics of cat predation and their comprehension would help to prevent or mitigate zoonotic risks. Systematic studies of cat predation on bats should include, in addition to behavioral observations, molecular assessments of the diet of cats and strict cooperation with wildlife rehabilitation centers, where bats injured by cats are often admitted [13]. Confirming that a given bat was killed or injured by a cat is today possible thanks to forensic DNA analysis techniques, which can even be used to link predation events to individual cats [32] and allow inspection of both predator and prey for the potential presence, or transmission, of pathogens. Estimating survival rates among bats following predation attempts from cats is also key to better evaluate the potential zoonotic risk associated with these events;
6. Since tools to explore epidemiological scenarios are available [106], including the use of GIS and spatial modeling, applications to situations involving people, cats and bats in rural and urban areas are needed;
7. Domestic cats are highly popular and many people, including animal lovers in good faith, keep cats outdoor to meet their pet’s supposed need for “freedom”. Cat impact on wildlife is often deliberately glossed over, generates animosity among cat lovers and is sometimes even exacerbated by a real “code of silence”. There is ever growing evidence, however, that cat predation on wildlife (e.g., [9,14,28]), including bats ([107]; this study), is largely underestimated. Awareness-raising campaigns should be carried out to encourage people to keep cats indoor, which would in fact protect not only wildlife from predation, but also cats from the many risks posed by living outdoors, and people from the zoonotic risks we discussed;
8. Finally, we urge that a stronger cooperation among potential stakeholders (conservationists, animal welfare organizations, human health authorities, virologists, etc.) is developed to implement appropriate one-health strategies and prevent or at least mitigate the risks of bat–cat–human transmission of pathogens.

## Figures and Tables

**Table 1:** Cases of bat predation by cats documented in the scientific literature. Cat classified as: F = feral; SF = semi-feral; O = owned; U = unknown. Habitat: H = human habitat such as agricultural or urban; N = natural habitat; U = unknown. International Union for Conservation of Nature (IUCN) status as follows: DD = Data Deficient; LC = Least Concern; NT = Near Threatened; VU = Vulnerable.

Family	Species	Cat Type	Sample	Country	Habitat	IUCN Status	Reference
Miniopteridae	*Miniopterus schreibersii*	U	Records of rescue	Italy	H	NT	[13]
		O	Survey	Italy	H		[14]
Molossidae	*Austronomus australis*	F	Stomach	Australia	N	LC	[15]
	*Mormopterus planiceps*	F	Stomach	Australia	H	LC	[16]
	*Tadarida brasiliensis*	U	Camera	Argentina	H	LC	[17]
	*Tadarida teniotis*	U	Records of rescue	Italy	H	LC	[13]
Mormoopidae	*Mormoops blainvillei*	F	Camera, scats and body remains	Puerto Rico	N	LC	[18]
	*Pteronotus quadridens*	F	Camera, scats and body remains	Puerto Rico	N	LC	[18]
Mystacinidae	*Mystacina tuberculata*	F	DNA and body remains	New Zealand	N	VU	[19]
		U	Survey		U		[20]
Natalidae	*Chilonatalus macer*	F	Scats	Cuba	N	DD	[21]
	*Natalus primus*	F	Scats	Cuba	N	VU	[21]
Phyllostomidae	*Artibeus lituratus*	O	Observations and body remains and rescue	Brazil	H	LC	[22]
		U	Observations	Brazil	U		[23]
	*Brachyphylla* *cavernarum*	F	Camera, scats and body remains	Puerto Rico	N	LC	[18]
	*Carollia perspicillata*	U	Observations	Brazil	U	LC	[23]
	*Desmodus rotundus*	O	Observations and body remains	Brazil	H	LC	[24]
	*Erophylla bombifrons*	F	Camera, scats and body remains	Puerto Rico	N	LC	[18]
	*Monophyllus redmani*	F	Camera, scats and body remains	Puerto Rico	N	LC	[18]
	*Phyllostomus discolor*	O	Observations, body remains and rescue	Brazil	H	LC	[22]
	*Phyllonycteris poeyi*	U	Observations of predator in the cave	Cuba	U	LC	[25]
		U	Scats	Cuba	N		[21]
	*Sturnira lilium*	U	Observations	Brazil	U	LC	[23]
	*Pteropus dasymallus*	F/SF	Survey	Japan	H	VU	[26]
	*Pteropus natalis*	F	Scats and stomach	Christmas Island	H	VU	[27]
	*Pteropus ornatus*	F	Scats	New Caledonian	N	VU	[28]
Pteropodidae	*Pteropus tonganus*	F	Scats	New Caledonian	N	LC	[28]
	*Pteropus vetulus*	F	Scats	New Caledonian	N	VU	[28]
	*Rousettus aegyptiacus*	O	Brought home	Israel	H	LC	[29]
	*Syconycteris australis*	O	Corpses brought	Australia	N	LC	[30]
Rhinolophidae	*Rhinolophus* *ferrumequinum*	U	Camera trap	Italy	H	LC	[13]
		O	Survey	Italy	H		[14]
	*Rhinolophus* *hipposideros*	U	Records of rescue	Italy	H	LC	[13]
		O	Survey	Italy	H		[14]
Vespertilionidae	*Chalinolobus gouldii*	F	Stomach	Australia	N	LC	[15]
		F	Stomach	Australia	H		[16]
		F	Stomach	Australia	H		[31]
	*Chalinolobus* *turbeculatus*	U	Survey	New Zealand	N and H	VU	[20]
	*Eptesicus serotinus*	U	Records of rescue	Italy	H	LC	[13]
		O	Survey	Italy	H		[14]
		U	Molecular analysis	United Kingdom	U		[32]
	*Hypsugo savii*	O	Records of rescue	Italy	H	LC	[13]
	*Myotis bechsteinii*	U	Records of rescue		H	NT	[13]
	*Myotis mystacinus*	O	Survey	Italy	H	LC	[14]
		U	Necropsy	Germany	U		[33]
		U	Molecular analysis	United Kingdom	U		[32]
	*Myotis crypticus*	U	Records of rescue	Italy	H	LC	[13]
		O	Survey	Italy	H		[14]
		U	Molecular analysis	United Kingdom	U		[32]
	*Myotis vivesi*	U	Scats	Mexico	N	VU	[34]
	*Nyctalus leisleri*	U	Records of rescue	Italy	H	LC	[13]
		O	Survey	Italy			[14]
	*Nyctalus noctula*	U	Body remains	Ukraine	H	LC	[35]
	*Nyctophilus geoffroyi*	F	Stomach	Australia	N	LC	[15]
		O	Corpses brought	Australia	H		[36]
		O	Corpses brought/Scats	Australia	H		[37]
		F	Scats	Australia	N		[38]
		F	Stomach	Australia	H		[39]
		F	Stomach	Australia	N		[40]
		F	Scats	Australia	N		[41]
	*Pipistrellus* *coromandra*	SF	Rescue and free	India	H	LC	[42]
	*Pipistrellus kuhlii*	O	Records of rescue	Italy	H	LC	[13]
	*Pipistrellus maderensis*	O	Corpses brought	Portugal	H	VU	[43]
	*Pipistrellus nathusii*	U	Records of rescue	Italy	H	LC	[13]
		O	Survey	Italy	H		[14]
		U	Necropsy	Germany	U		[33]
	*Pipistrellus pipistrellus*	O	Records of rescue	Italy	H	LC	[13]
		U	Wing damage and molecular analyses	United Kingdom	U		[32,44]
		U	Necropsy	Germany	H		[33]
	*Pipistrellus pygmaeus*	O	Records of rescue	Italy	H	LC	[13]
		U	Wing damage and molecular analysis	United Kingdom	U		[32,44]
	*Plecotus auritus*	U	Records of rescue	Italy	H	LC	[13]
		O	Survey	Italy	H		[14]
		U	Necropsy	Germany	H		[33]
		U	Molecular analysis	United Kingdom	U		[32]
	*Vespadelus darlingtoni*	O	Corpses brought	Lord Howe Island	N/U	LC	[45]
	*Vespertilio murinus*	U	Necropsy	Germany	U	LC	[33]
